# A simple formula for predicting the warfarin dose in atrial fibrillation: development, external validation, and model comparison

**DOI:** 10.1186/s12959-025-00776-y

**Published:** 2025-10-09

**Authors:** Anunya Ujjin, Natnicha Pongbangli, Wanwarang Wongcharoen, Arisara Suwanagool, Chatree Chai-adisaksopha

**Affiliations:** 1 Division of Medicine, Neurological Institute of Thailand, Bangkok, Thailand; 2 Division of Cardiology, Department of Internal Medicine, Chiang Rai Prachanukroh Hospital, Chiang Rai, Thailand; 3 Division of Cardiology, Department of Internal Medicine, Faculty of Medicine, Chiang Mai University, Chiang Mai, Thailand; 4 Division of Cardiology, Department of Medicine, Faculty of Medicine Siriraj Hospital, Mahidol University, Bangkok, Thailand; 5 Department of Internal Medicine, Faculty of Medicine, Chiang Mai University, Chiang Mai, Thailand; 6 Department of Internal Medicine, Sakaeo Crown Prince Hospital, Sakaeo, Thailand

**Keywords:** Warfarin, Atrial fibrillation, Formula

## Abstract

**Background:**

The dose of warfarin varies between individuals. Several formulas for predicting the maintenance dose of warfarin have been developed; however, most are complicated and not practical for clinical use.

**Objective:**

To determine factors that predict warfarin dosage and the relationship between clinical variables and the maintenance dose of warfarin, and to develop a simple formula for predicting the maintenance dose of warfarin that is particularly useful for identifying patients with atrial fibrillation (AF) who are at higher risk of bleeding, without relying on pharmacogenetic data.

**Materials and methods:**

This was a retrospective cohort study carried out between 2011 and 2021. All patients are started on warfarin with a target INR of 2.0 to 3.0. The prediction models for the maintenance dose were developed using a first-order equation. Correlation and performance of the formula were examined in training and validation cohorts.

**Results:**

A training cohort consisted of 520 patients with a mean age of 69 ± 12 years. The proposed warfarin dosing formula was 3+(0.02×body weight (kg))-(0.02×age (years))-(0.4×serum creatinine (mg/dL)).When compared with a warfarin dosing formula, a 3-mg dose was associated with overdosing with an odds ratio [OR] of 3.31 (95%CI 2.26–4.84, *p* < 0.0001) in patients whose body weight was < 60 kg, OR 3.08 (95%CI 2.15–4.40, *p* < 0.0001) in patients aged ≥ 70 years and OR 2.39 (95% CI 1.67–3.44, *p* < 0.0001) in patients with eGFR < 50 mL/min. The findings in the validation cohort of 632 patients were concordant with the training cohort.

**Conclusion:**

A simple warfarin dosing formula incorporating age, body weight, and serum creatinine reduced the risk of warfarin overdose in a high-risk population.

**Supplementary Information:**

The online version contains supplementary material available at 10.1186/s12959-025-00776-y.

## Introduction

In comparison with control or placebo, vitamin K antagonist (VKA) therapy reduces stroke risk by 64% and mortality by 26% [1] in patients with atrial fibrillation (AF). Therefore, VKA remains a significant oral anticoagulant therapy for the prevention of stroke and systemic embolism in AF patients. Moreover, VKA is currently the only treatment with established safety values in AF patients with rheumatic mitral valve disease and/or an artificial heart valve [2].

There is a steadily growing trend in the use of a direct oral anticoagulant (DOAC) (from 4.7 to 47.9%) over time, while warfarin usage has declined (from 52.4–17.7% [3]. Nevertheless, warfarin remains a commonly used oral anticoagulant in circumstances where DOAC is not indicated.

The observed bleeding rate caused by incorrect warfarin dosing has been reported to be 10–16% per year. Personalized warfarin dosing based on clinical and genetic information is a potential solution to this issue. When warfarin treatment is initiated, the optimal dose for the therapeutic range is mainly established through trial and error. Fluctuation of the INR is frequently observed, especially in the early phase after the initiation of warfarin [4]. Such fluctuations increase the risk of bleeding and thromboembolism [5]. Therefore, the continuous measurement of the prothrombin time with the international normalized ratio (INR) is necessary for adjustment to find the most effective dose of warfarin [6].

Due to its narrow therapeutic index and inter-patient variability in dose requirement, this drug has been considered an ideal target for personalized medicine. Several warfarin dosing algorithms have been proposed to tailor the warfarin dosage in various populations, including European, Asian, and African-American groups [7–10]. Within Asian populations, existing models have often integrated pharmacogenetic variables, as demonstrated by studies from Ichihara et al. [7] However, despite these advancements, there remain fewer simplified, clinically applicable models developed specifically for Asian populations that do not require pharmacogenetic data [8]. Factors influencing warfarin dose requirements may differ across geographic regions and ethnic groups, underscoring the ongoing need for diverse and population-specific dosing strategies, including those readily implementable in varied clinical settings.

As of 2024, major clinical guidelines acknowledge the importance of genetic factors in warfarin dosing but do not recommend routine pharmacogenetic testing for all patients. The Clinical Pharmacogenetics Implementation Consortium (CPIC) provides detailed recommendations for genotype-guided dosing based on *VKORC1* and *CYP2C9* variants when genetic information is available, yet it stops short of endorsing universal testing [9]. Similarly, the American College of Chest Physicians (ACCP) 2021 guidelines advise against routine genetic testing before initiating warfarin, citing insufficient evidence that such testing improves clinical outcomes [10–12]. The U.S. Food and Drug Administration (FDA) also recommends considering genetic factors during dosing decisions but does not mandate genetic testing for all patients.

This study aimed to identify the clinical factors influencing warfarin maintenance dose and to develop a simple formula for predicting the warfarin dose in atrial fibrillation patients without using genetic testing. The study also includes external validation and comparison with existing dosing models.

## Patients and methods

### Sampling and data collection

#### Training cohort

The training cohort comprised of participants ≥ 15 years of age who were diagnosed with AF and had been receiving warfarin with a target INR of 2.0 to 3.0 at the outpatient clinic, Sakaeo Crown Prince Hospital, Sakaeo, Thailand. Exclusion criteria included patients in whom an INR of 2.0 to 3.0 had not been achieved in at least two consecutive follows-ups, and those who had mechanical valves which may need a higher target INR. Patients were also excluded if there were missing essential data regarding body weight, serum creatinine, INR or dose of warfarin. We collected the following patient information, age, body weight, comorbidities, the primary indication for warfarin treatment, the maintenance dose of warfarin, CHA_2_DS_2_-VASc Score, initial serum creatinine (mg/dL), the use of concomitant drugs known to have potential interaction with warfarin, for example amiodarone, and bleeding complication. The study was conducted following the ethical principles of the Declaration of Helsinki (revised in 2013). The study protocol was approved by the Medical Ethics Committee of Sakaeo Crown Prince Hospital (project code S003b/65 ExPD) and Chiang Mai University (project code MED-2566-0130).

#### Validation cohort

The validation cohort consisted of participants ≥ 15 years of age who were diagnosed with AF and had been receiving warfarin with a target INR of 2.0 to 3.0 at the outpatient clinic, Maharaj Nakorn Chiang Mai Hospital, Chiang Mai, Thailand.

Patients were excluded if an INR 2.0 to 3.0 had not been achieved for at least two consecutive follow-ups during the first year of warfarin therapy and those who had incomplete data. Data on gender, weight, serum creatine and actual maintenance warfarin dose were collected.

### Measurement and outcomes

The study focused on two outcome measures: the actual maintenance warfarin dose and the predicted warfarin dose. The actual maintenance warfarin dose was defined as the dosage that resulted in an INR of 2.0 to 3.0 for two consecutive measurements. Subsequently, we investigated the relationship between clinical variables and the actual warfarin dose. Based on this analysis, we developed a formula to predict the maintenance dose of warfarin using logistic regression analysis (predicted warfarin dose).

The primary objective of the study was to determine the percentage of patients who received an appropriate warfarin dose. An appropriate dose was defined as the predicted dose of warfarin falling within a range of −20% to + 20% of the actual maintenance dose for each patient. If the predicted dose was lower than the actual maintenance dose, it was considered an underdosing scenario. Conversely, if the predicted dose exceeded the actual dose, it was classified as overdosing.

Following that, we conducted a comparison between two strategies for predicting warfarin dosage: the formula-based approach and assigned-dosing regimens. Assigned-dosing regimen was defined as the starting warfarin dose was assigned without the use of a formula. In this study we evaluated various assigned-dosing regimens (2 mg, 2.5 mg, 3 mg, 3.5 mg,4 mg and 5 mg) and compared their outcomes to those predicted by the formula. The goal was to identify the most effective regimen that would yield the highest proportion of appropriate warfarin doses. In particular, we sought to minimize both underdosing and overdosing.

### Statistical analyses

Statistical analysis was performed using STATA Statistical software version 16. Continuous variables were tested for normality using histograms and normal density plots. Normally distributed continuous data are described with mean and standard deviation while non-normally distributed data were described with median and interquartile range. Categorical data were described with frequency and percentage. For univariable comparison of continuous variables, an independent t-test or rank sum test were used as appropriate. For comparison of categorical variables, Fisher’s exact probability test was used.

All clinical parameters for predicting warfarin dosage are presented using descriptive statistics. We displayed the relationship between age, body weight, creatinine clearance and the maintenance dose of warfarin using a scattergram. From these data we acquired a prediction formula for approximation of a maintenance dose of warfarin.

Pearson’s correlation test was performed to analyze the linear correlation between predicted dose and actual dose of warfarin. We employed multivariate linear regression analysis to investigate the association between warfarin dose and clinical variables. Subsequently, we developed a novel warfarin-dosing algorithm to predict warfarin maintenance dose. We assessed the performance of the new formula with a warfarin predicting formula previously published in a relevant study [8], and a various fix-dosing regimens. A statistically significant difference was defined as a p-value less than 0.05.

## Results

### Training cohort

**Fig. 1:**
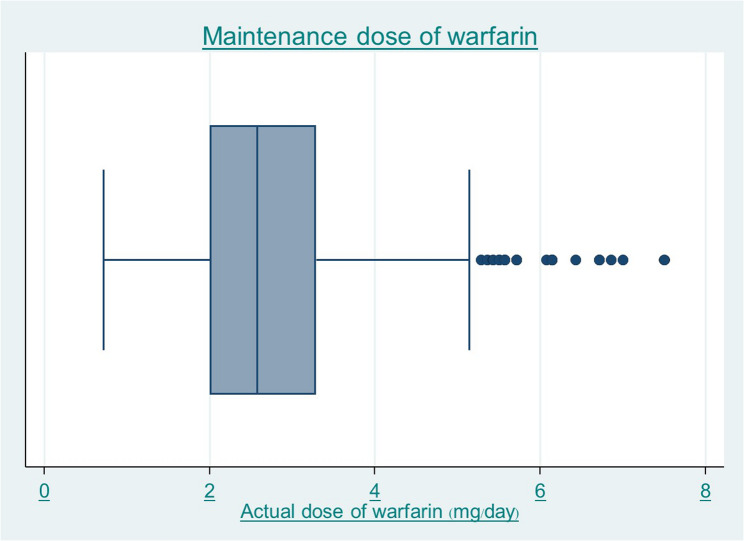
The maintenance dose of warfarin (mg/day)

A total of 520 Thai patients were eligible for inclusion in the study. Demographic data are summarized in Table 1. In brief, the mean age was 69 ± 12 years, the proportion of females being 55.6% (*n* = 289). Mean body weight was 61.5 ± 14 kg. CHA_2_DS_2_-VASc Scores 3 to 5 encompassed 74.8% of patients. The maintenance warfarin dose was 2.76 ± 1.16 mg/day, Fig. 1.

**Table 1:** Patient characteristics

**Baseline characteristics**	**Training cohort** **(n=520)**	**Validation cohort** **(n=632)**
Age (years)		
Mean ± SD	69 ± 12	65 ± 13
Median (IQR)	70 (61, 78)	66 (56, 76)
< 70 n (%)	253 (48.6)	382 (60.4)
≥ 70 n (%)	267 (51.4)	250 (39.6)
Female n (%)	289 (55.6)	355 (56.2%)
Body weight (kg)		
Mean ± SD	61.5 ±14	59.2 ±15
Median (IQR)	60 (52, 70)	58 (50, 67)
< 60 n (%)	249 (47.9)	351 (55.5)
≥ 60 n (%)	271 (52.1)	281 (44.5)
Serum creatinine (mg/dL)		
Mean ± SD	1.03 ± 0.43	1.16 ± 0.85
Median (IQR)	0.92 (0.78, 1.15)	1 (0.8, 1.2)
Estimated creatinine clearance (mL/min)		
Mean ± SD	57.9 ± 30.4	NA
Median (IQR)	51 (31,71)	NA
CHA_2_DS_2_-VASc Score (n=393)		
0 n (%)	1 (0.3)	NA
1 n (%)	12 (3.1)	
2 n (%)	59 (15.0)	
3 n (%)	114 (29.0)	
4 n (%)	104 (26.5)	
5 n (%)	76 (19.3)	
6 n (%)	22 (5.6)	
7 n (%)	5 (1.3)	
Co-morbid diseases^a^ n (%)		
Diabetes mellitus	122 (23.5)	115 (18.0)
Hypertension	300 (57.7)	301 (47.0)
Congestive heart failure	191 (36.9)	103 (16.1)
Cirrhosis	13 (2.5)	5 (0.8)
Chronic kidney disease	NA	96 (15)
ESRD^b^ on RRT^c^	2 (0.4)	NA
Ischemic stroke	179 (34.4)	109 (17.0)
Medication n (%)		
Statin	313 (60.2)	NA
Amiodarone	2 (0.4)	NA

*NA *Not applicable

^a﻿^In the Validation cohort, the data is from the original paper (n=640)

^b^ESRD = end-stage renal disease

^c^RRT = renal replacement therapy

### Relationship between clinical variables and warfarin dose

**Fig. 2:**
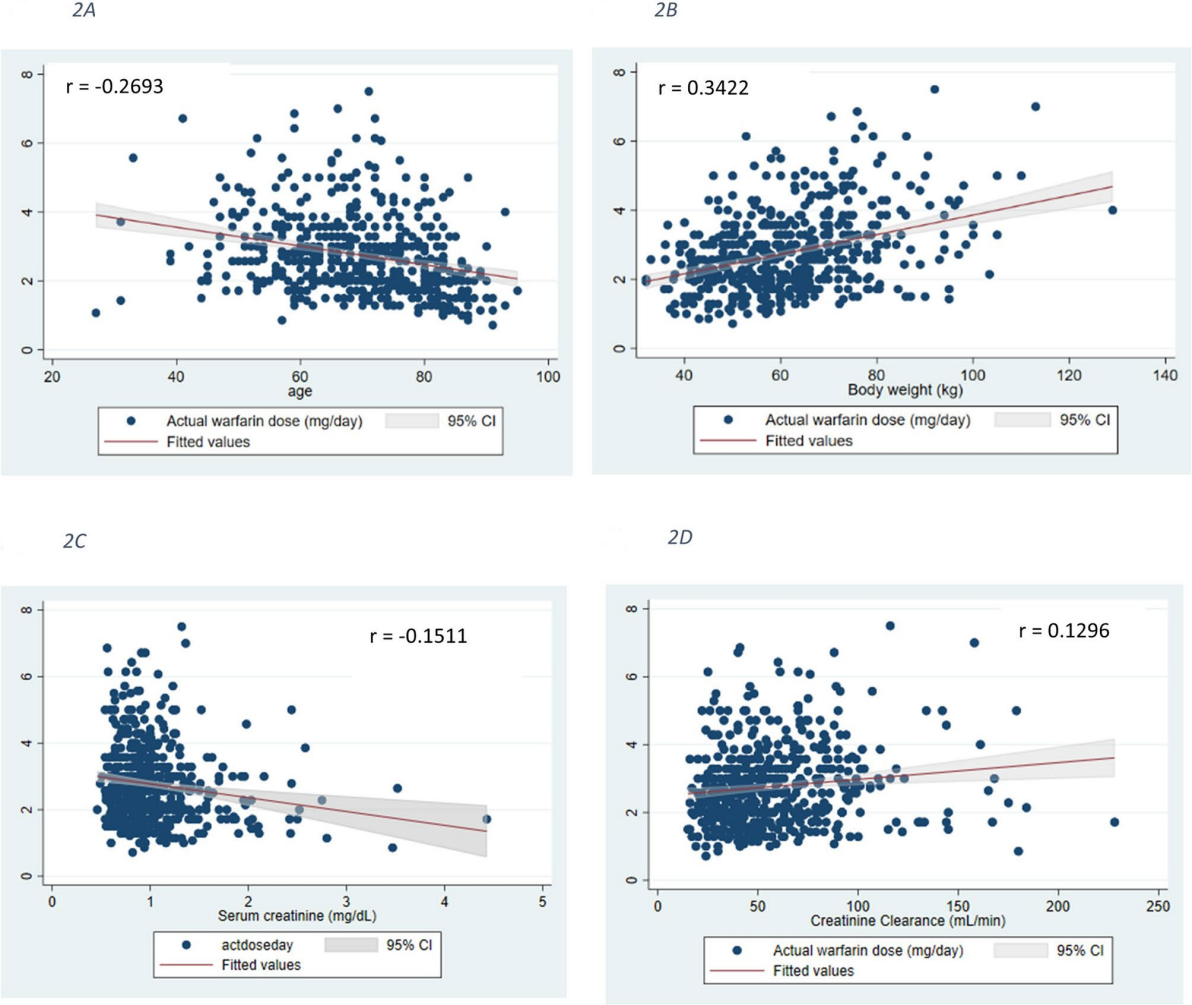
The correlation between age (years) (**A**), body weight (kg) (**B**), serum creatinine (mg/dL) (**C**), creatinine clearance (mL/min) (**D**) and the maintenance dose of warfarin (mg/day)

 We analyzed the relationship between various clinical variables and the maintenance dose of warfarin. The correlation between the age, body weight and creatinine clearance and the maintenance dose of warfarin is shown in Fig. 2. The simple linear regression models showed that age and serum creatinine (mg/dL) had a negative correlation with warfarin dose, *r* = 0.2714, *p* < 0.001 and *r* = 0.1544, *p* = 0.004, respectively, whereas body weight (kg) had a positive correlation with warfarin dose, *r* = 0.3498, *p* < 0.001. A stepwise multivariate regression model was performed to determine the predicted formula for warfarin dose. We included parameters with *p* < 0.20 in the univariate analysis as candidates for the multivariable analysis. Additionally, we considered previous postulates for predicting warfarin dose, such as the presence of heart failure and/or a history of stroke. We employed a two-sided *p* < 0.05 as the threshold for statistical significance. It is worth mentioning that we specifically excluded the CHA2DS2-VASc Score from the multivariable analysis due to its inability to explain scientific relationships and its collinearity with age. Considering the overlapping information between age and the CHA2DS2-VASc Score, we decided to exclude the latter from the multivariable model to avoid multicollinearity issues and maintain the interpretability of the final formula.

The results of the univariable and multivariable linear regression analyses for the maintenance dose are shown in Table 2. In the multiple regression model, age, body weight and creatinine were independently associated with the maintenance dose of warfarin with beta-coefficients of −0.015, *p* = 0.001, 0.025, *p* < 0.001 and − 0.373, *p* = 0.002, respectively.

**Table 2:** Factors associated with maintenance dose of warfarin

Factors	β coefficient (per mg/day)	*p*-value		β coefficient (per mg/day)	*p*-value
**Univariable Gaussian regression analysis**			**Multivariable Gaussian regression analysis**		
Age (years)	−0.027	0.000*	Age	−0.015	0.001*
Body weight (Kg)	0.028	0.000*	Body weight	0.025	0.000*
Female	−0.110	0.283	Creatinine (mg/dL)	−0.373	0.002*
Creatinine (mg/dL)	−0.417	0.000*			
CHA_2_DS_2_-VASc Score	−0.132	0.005*			
Rheumatic mitral stenosis	−0.062	0.614			
Hemodialysis	0.019	0.982			
Liver cirrhosis	−0.454	0.164			
Heart failure	−0.128	0.228			
Ischemic stroke	0.043	0.683			

*p < 0.05

### Model development and validation of a formula for predicting warfarin dose

**Fig. 3:**
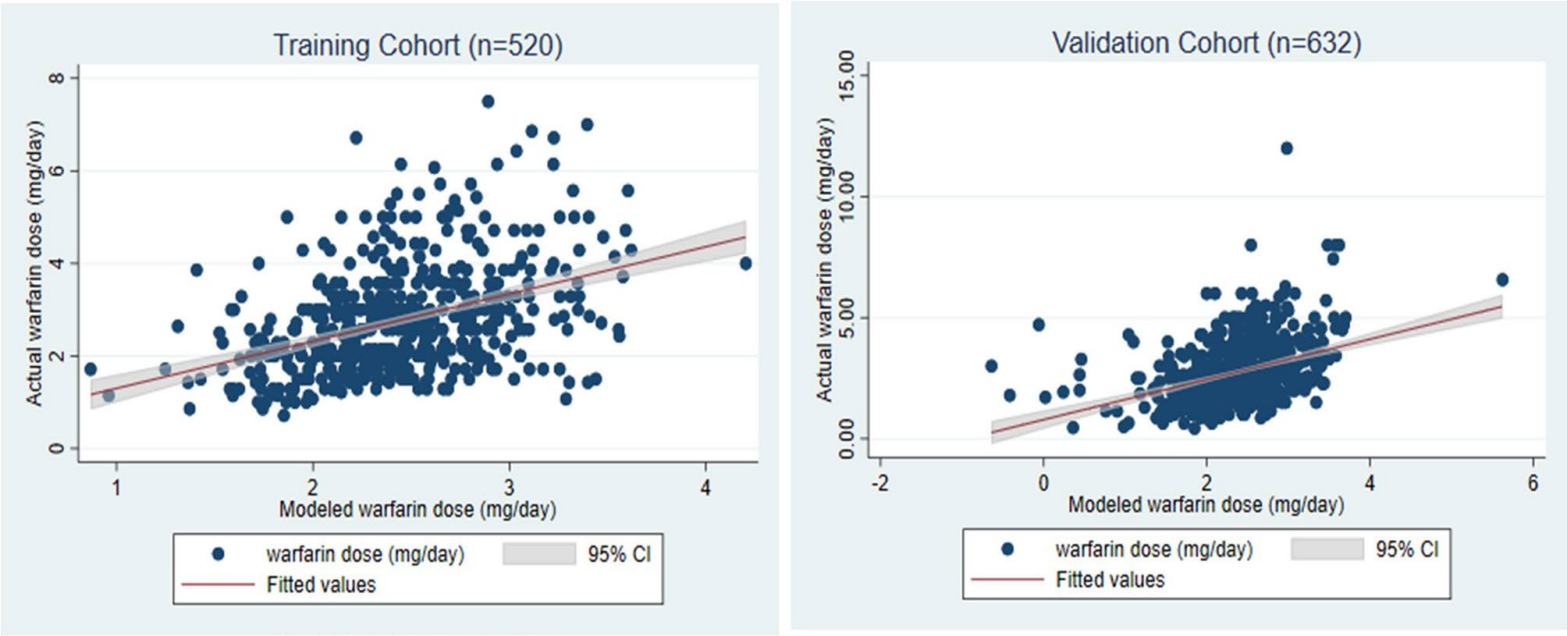
Correlation of the actual warfarin dose and the modeled warfarin dose, **A** Training cohort, **B** Validation cohort, Gray area indicates 95% confidence interval

We developed a linear formula aiming to predict the maintenance dose of warfarin by incorporating age, body weight and serum creatinine in the model. The multivariable Gaussian regression analysis indicated that age, body weight and serum creatinine were significantly associated with the maintenance dose of warfarin. We generated a simple correlation with a first-order approximation as follows: warfarin dose = 3 + (0.02 × body weight (kg))-(0.02 × age(years))-(0.4 × serum creatinine(mg/dL)). Pearson’s correlation analysis showed a significant correlation between the calculated warfarin dose using the warfarin dosing algorithm and actual dose, in the training cohort, and validation cohort, specifically *r* = 0.4056; *p* < 0.001 (Fig. 3).

### Comparison of the performance of warfarin dosing strategies

 We conducted a comparative analysis of different warfarin dosing strategies, including the new formula implemented in this study; warfarin dose (mg/day) = 3 + (0.02 × body weight (kg))-(0.02 × age(years))-(0.4 × serum creatinine(mg/dL)), a previous warfarin dosing formula; warfarin dose (mg/day) = 3.2 - (0.03 × age (years)) + (0.02 × body weight (kg)) (10% dose reduction if the presence of heart failure (HF) and/or stroke), and multiple assigned-dose regimens; 2-mg dose, 2.5-mg dose, 3-mg dose, 3.5-mg dose, 4-mg dose and a 5-mg dose to calculated the probability of over and underdosing compare in cases (Table 3). The new formula resulted in the highest proportion of patients who were classified into the appropriate dose group (39.2%), followed by a 3-mg dose (38.7%), the previous warfarin dosing formula (37.1%), the 2.5-mg dose (35.8%), the 2-mg dose (30.8%), the 3.5-mg dose (29.6%), the 4-mg dose (19.4%) and the 5-mg dose (10.8%), *p* < 0.001. The 4-mg dose and the 5-mg dose were associated with a higher number of patients who presented as overdosing (76.5% and 88.1%, respectively) compared to the new formula and the previous formula (27.3% and 18.7%, respectively).

**Table 3:** Performance of warfarin dosing strategies in classifying patients into appropriate dose, underdose, and overdose categories based on observed maintenance dose

	New formula^a^ (*n* = 520)	Previous warfarin dosing formula^b^ (*n* = 520)	2-mg dose (*n* = 520)	2.5-mg dose (*n* = 520)	3-mg dose (*n* = 520)	3.5-mg dose (*n* = 520)	4-mg dose (*n* = 520)	5-mg dose (*n* = 520)
Underdosing	174 (33.5%)	230 (44.2%)	287 (55.2%)	159 (30.6%)	90 (17.3%)	52(10.0%)	21(4.0%)	6 (1.2%)
Appropriate dose	204 (39.2%)	193 (37.1%)	160 (30.8%)	186 (35.8%)	201 (38.7%)	154(29.6%)	101(19.4%)	56 (10.8%)
Overdosing	142 (27.3%)	97 (18.7%)	73 (14.0%)	175 (33.7%)	229 (44.0%)	314(60.3%)	398(76.5%)	458 (88.1%)

Appropriate dose was defined as a predicted dose within±20%of the observed maintenance dose. Underdosing and overdosing were defined as predicted doses more than 20%below or above the observed dose, respectively

^a^New formula: Predicted dose=3+ (0.02×body weight (kg))−(0.02×age(years))−(0.4×serum creatinine(mg/dL))

^b^Previous warfarin dosing formula: predicted dose=3.2− (0.03×age (years)) + (0.02×body weight (kg)) (10%dose reduction if the presence of heart failure (HF) and/or stroke)

### Clinical relevance and risk of under- or over-dosing

Table 4 shows the performance of the new formula versus the 3-mg dose when classifying patients according to age, body weight and estimated creatinine clearance. The administration of a 3-mg dose of warfarin to patients aged over 70 years, with a body weight less than 60 kg, and an estimated creatinine clearance of less than 50 mL/min has been associated with an increased risk of overdose. When compared to a new formula, 3- mg dose was associated with increased risk of overdosing with the ORs of 3.08 (95% CI 2.15 to 4.40, *p* < 0.0001) for patients aged over 70 years, 3.31 (95% CI 2.26 to 4.84, *p* < 0.0001) for those with a body weight less than 60 kg, and 2.39 (95% CI 1.67 to 3.44, *p* < 0.0001) for those with an estimated creatinine clearance of less than 50 mL/min.

**Table 4:** Performance of the new formula versus the assigned 3-mg dose. Subgroup analysis according to age, body weight and estimated creatinine clearance with maintenance of warfarin dose

	Suboptimal dose		Optimal dose		Overdose	
	New formula	3-mg dose	New formula	3-mg dose	New formula	3-mg dose
Age(years)						
< 70	80(31.6%)	58(22.9%)	112(44.3%)	119(47.0%)	61(24.1%)	76(30.0%)
≥ 70	94(35.2%)	32(12.0%)	92(34.5%)	82(30.7%)	81(30.3%)	153(57.3%)
BW(Kg)						
< 60	84(33.7%)	23(9.2%)	104(41.8%)	97(39.0%)	61(24.5%)	129(51.8%)
≥ 60	90(33.2%)	67(24.4%)	100(36.9%)	104(38.4%)	81(30.0%)	100(36.9%)
eCrCl (mL/min)						
< 50	84(32.6%)	32(12.4%)	98 (38.0%)	97 (37.4%)	76(29.5%)	129(50.0%)
≥ 50	90(34.4%)	58(22.1%)	106(40.5%)	104(39.7%)	66(25.2%)	100(38.2%)

### Validation cohort

Of the 640 eligible patients at outpatient clinic, Maharaj Nakorn Chiang Mai Hospital, Chiang Mai, Thailand, 8 patients were excluded due to missing serum creatinine values. The remaining 632 patients were included in the analysis with a mean age of 65 ± 13 years, 56.2% were female, mean body weight was 59 ± 15 kg and mean serum creatinine was 1.2 ± 0.85 mg/dL. Clinical, demographic, and co-morbid diseases are presented in Table 1. The maintenance warfarin dose was 2.79 ± 1.25 mg/day.

The findings in the validation cohort were concordant with the training cohort. Pearson’s correlation analysis showed a significant correlation between the calculated warfarin dose using the warfarin-dosing algorithm and actual dose, *r* = 0.3896; *p* < 0.001 (Fig. 3B).

The new formula had the highest proportion of patients who were classified into the appropriate dose group (42.1%), followed by the previous warfarin dosing formula (40.9%), the 3-mg dose (39.1%), the 2.5-mg dose (36.6%), the 2-mg dose (35.4%), the 3.5-mg dose (33.5%), the 4-mg dose (23.9%), and the 5-mg dose (10.1%), *p* < 0.001. Additionally, the 4-mg dose and the 5-mg dose were associated with a higher number of patients who were identified as overdosing (72.5% and 88.6%, respectively) compared to the new formula and the previous formula (24.1% and 20.9%, respectively), Supplementary Table 1.

The subgroup analyses of age, body weight and estimated creatinine clearance with maintenance of warfarin dose are presented in supplementary Table 2. The administration of a assigned 3-mg dose of warfarin to patients aged over 70 years, with a body weight less than 60 kg, and an estimated creatinine clearance of less than 50 mL/min has been associated with an increased risk of overdose. When compared to a new formula, 3 mg-assigned dose was associated with increased risk of overdosing with the ORs of 4.65 (95% CI 3.16 to 6.84, *p* < 0.0001) for patients aged over 70 years, 3.20 (95% CI 2.32 to 4.39, *p* < 0.0001) for those with a body weight less than 60 kg, and 4.54 (95% CI 3.10 to 6.64, *p* < 0.0001) for those with an estimated creatinine clearance of less than 50 mL/min.

## Discussion

This study demonstrated an association between age, body weight and renal function with a maintenance dose of warfarin. We developed a new formula, incorporating age, body weight and serum creatinine, and age to predict a maintenance dose of warfarin. Our decision to include body weight over body mass index (BMI) was based on a comparative multivariable regression analysis within our cohort. This analysis revealed that a model incorporating age, body weight, and serum creatinine yielded a higher R² (0.16) compared to a model using age, BMI, and serum creatinine (R² = 0.11), indicating superior predictive value for body weight. This finding is also consistent with prior studies that have identified body weight as a more robust predictor of warfarin dose than BMI [7, 8].

Within Asian populations, existing models have often integrated pharmacogenetic variables. For instance, studies by Ichihara et al. in Japan have significantly contributed to understanding the genetic determinants of warfarin dosing in Japanese patients, notably including the VKORC1 −1639 G > A polymorphism as an independent variable [7]. In China, a multicenter randomized controlled trial demonstrated that genotype-guided dosing was superior to standard clinical dosing, using the percentage of time in the therapeutic range as the primary outcome [13].

Despite acknowledging the influence of genetic factors such as VKORC1 and CYP2C9, major clinical guidelines (e.g., CPIC, FDA, and ACCP 2021) [9, 11, 12, 14] do not recommend routine pharmacogenetic testing for all warfarin patients, citing insufficient evidence of improved clinical outcomes. In Thailand, warfarin is prescribed under the Universal Coverage scheme, but there are no clinical guidelines recommending genetic testing. Therefore, we conducted this research to provide physicians with guidance on warfarin dosing without relying on genetic information.

The optimal maintenance dose of warfarin can vary widely among individual patients. To investigate the relationship between clinical parameters and the required maintenance dose of warfarin, several studies have been conducted. One study found that patients aged over 65 required significantly lower doses of warfarin (25.4 ± 11.5 mg/week) compared to patients aged 45–65 years (32.8 ± 15.7 mg/week) and those aged 18–44 (39.7 ± 18.6 mg/week). Additionally, it demonstrated that obese patients required a higher dose of warfarin (32.2 ± 15.2 mg/week) than those with a normal weight (27.4 ± 17.3 mg/week) [15]. With regard to kidney function, patients with moderate renal impairment required doses 9.5% lower than those with minimal or mild impairment, while patients with severe renal impairment required doses 19% lower [16]. A previous study showed that the maintenance warfarin dose was positively correlated with kidney function as represented by eCrCl in Japanese patients [7]. Incorporating eCrCl into the dosage calculation improved the accuracy of predicting the maintenance dose of warfarin in this population. These findings were consistent with the present study, emphasizing that patients who are older, have impaired renal function, or low body weight may require lower doses of warfarin.

We developed a simple formula to predict the maintenance dose of warfarin based on clinical variables identified through the multiple regression analysis: warfarin dose (mg/day) = 3 + (0.02 x body weight (kg)) – (0.02 x age (years)) – (0.4*serum creatinine (mg/dL)). In both the training and validation cohorts, the predicted warfarin dose showed a moderate correlation with the actual warfarin dose. Our study compared multiple strategies for warfarin dosage, including a previous warfarin dosing formula [8], 2-mg, 2.5-mg, 3-mg dose, 3.5-mg dose, 4-mg dose and 5-mg dose. The predicted warfarin dose calculated using our formula resulted in a higher number of patients falling in the appropriate dose categories in both the training and validation cohorts (39.2% and 42.1%, respectively). Importantly, there was a significant reduction in the number of patients who would receive an excessive dose of warfarin.

The proportion of overdosing appears moderately higher with the new formula compared to the past formula (27.3% vs. 18.7%). Nevertheless, we found that the past formula tended to underestimate warfarin dose, resulting in a higher rate of under-dosing (44.2% vs. 33.5%) when compared to the new formula. This suggests that the past formula had a higher propensity for suboptimal dosing, potentially leading to decreased therapeutic efficacy in a significant portion of patients.

Furthermore, it is crucial to consider the proportion of appropriate dosing, which is an important measure of the formula’s effectiveness. In this regard, the new formula demonstrated a higher rate of appropriate dosing compared to the past formula (39.2% vs. 37.1%). This indicates that the new formula achieved a more favorable balance between under-dosing and over-dosing, resulting in a greater proportion of patients receiving appropriate and effective warfarin doses.

Another approach to predicting the maintenance warfarin dose involved the early INR response. A single-staged predicting formula that relies on early INR values has been developed and has shown an acceptable predicting accuracy (R^2^ = 0.706) [17].

It is important to note that the warfarin dosing formula in this present study did not incorporate genetic information of individual patients. In recent years, a pharmacogenetics-based approach has emerged [18–21]. A previous study showed that a dosing algorithm incorporating point-of-care genotyping information led to improved anticoagulation control. However, the genetic testing for warfarin dosing is not routinely recommended in general clinical practice guidelines nowadays [22]. The genetic testing is also costly and is not widely available in clinical practice.

One of advantages of the warfarin dosing formula developed in this study is its ability to prevent overdosing in high-risk patients, including those with low body weight, old age or renal impairment. A subgroup analysis of patients with these risk factors demonstrated an increased risk of overdosing when 3-mg dose regimen was used instead of the dose predicted by the formula. This finding highlights the potential role of using this warfarin dosing formula in patients with high-risk warfarin overdose.

The strength of this present study is that we developed a warfarin dosing formula based on a large cohort of patients with AF who were initiates of warfarin for the prevention of stroke and systemic embolism. In addition, the study population was relatively homogenous in terms of genetic background. Secondly, we examined the validity of the warfarin dosing formula using the validation cohort of AF patients, and the findings were consistent with those of the training cohort, indicating that the warfarin dosing formula is valid in Thai patients. Our study had several limitations. First, the new formula for this warfarin dosing formula may not be applicable in patients with a target INR greater than 2.0 to 3.0. Moreover, the findings of this study may limit the generalizability in Asian population due to genetic variation. It is noteworthy that Asian patients typically have a lower body weight compared to Caucasian patients, which may further impact the applicability of the study’s results. Lastly, to enhance the formula’s generalizability, we did not include some potential variables such as hepatic function, lipid profile, and B-type natriuretic peptide that could potentially affect the warfarin dosage. In the future, as pharmacogenomic testing becomes more widely available and integrated into personalized medicine, we recognize that genetic factors, particularly VKORC1 and CYP2C9 could further enhance the predictive accuracy and clinical utility of warfarin dosing models.

## Conclusion

We developed a new formula for predicting the maintenance dose of warfarin using body weight, age, and creatinine (mg/dL). The findings of this study suggest that the use of this formula is effective in preventing overdosing in comparison to 3-mg dose, particularly in patients with high-risk features for warfarin overdose. These results highlight the potential clinical value of using our formula for warfarin dosing in certain patient populations.

## Supplementary Information

Supplementary Material 1.

## Data Availability

Data Availability Statement: The data that support the findings of this study are available on request from the corresponding author, [C.C.]. The data are not publicly available due to restriction of ethics’ policy.
