# Supplementary material for: A simple formula for predicting the warfarin dose in atrial fibrillation: development, external validation, and model comparison
Source: Thromb J. 2025 Oct 9;23:96. doi: 10.1186/s12959-025-00776-y (PMC12512375; doi:10.1186/s12959-025-00776-y)
Supplement: Supplementary file 1 — Supplementary Material 1. [file 12959_2025_776_MOESM1_ESM.docx]

**Supplementary table**

***Validation cohort***

**Supplemental Table 1.** Performance of warfarin dosing strategies in classifying patients into appropriate dose, underdose, and overdose categories based on observed maintenance dose.

|  | **New formula§**  **(n=632)** | **Previous warfarin dosing formula§§**  **(n=640)** | **2 mg dose**  **(n=632)** | **2.5 mg dose**  **(n=632)** | **3-mg dose**  **(n=632)** | **3.5-mg dose**  **(n=632)** | **4-mg dose**  **(n=632)** | **5-mg dose**  **(n=632)** |
| --- | --- | --- | --- | --- | --- | --- | --- | --- |
| **Underdosing** | 214(33.9%) | 244(38.1%) | 317(50.2%) | 199(31.5%) | 113 (17.9%) | 59 (9.3%) | 23 (3.6%) | 8(1.3%) |
| **Appropriated dose** | 266 (42.1%) | 262 (40.9%) | 224(35.4%) | 231(36.6%) | 247 (39.1%) | 212(33.5%) | 151(23.9%) | 64(10.1%) |
| **Overdosing** | 152 (24.0%) | 134 (20.9%) | 91(14.4%) | 202(32.0%) | 272 (43.0%) | 361(57.1%) | 458(72.5%) | 560(88.6%) |

**^§New formula: Predicted dose =^** ^3 + (0.02 × body weight (kg)) - (0.02 × age(years)) - (0.4 × serum creatinine(mg/dL))^

**^§§ Previous warfarin dosing formula: predicted dose =^** ^3.2- (0.03 × age (years)) + (0.02 × body weight (kg)) (10% dose reduction if the presence of heart failure (HF) and/or stroke).^

^Appropriate dose was defined as a predicted dose within ±20% of the observed maintenance dose. Underdosing and overdosing were defined as predicted doses more than 20% below or above the observed dose, respectively.^

**Supplemental Table 2.** Performance of the new formula versus the assigned 3-mg dose. Subgroup analysis according to age, body weight and estimated creatinine clearance with maintenance of warfarin dose.

|  | **Suboptimal dose** | | **Optimal dose** | | **Overdose** | |
| --- | --- | --- | --- | --- | --- | --- |
|  | **New formula** | **3-mg**  **dose** | **New formula** | **3-mg**  **dose** | **New formula** | **3-mg**  **dose** |
| Age(years)  < 70  ≥ 70 | 119(31.2%)  97(38.8%) | 88(23.0%)  25(10.0%) | 169(44.2%)  95(38.0%) | 168(44.0%)  79(31.6%) | 94(24.6%)  58(23.2%) | 126(33.0%)  146(58.4%) |
| BW(kg)  < 60  ≥ 60 | 126(35.9%)  90(32.0%) | 44(12.5%)  69(24.6%) | 135(38.5%)  129(45.9%) | 123(35.0%)  124(44.1%) | 90(25.6%)  62(22.1%) | 184(52.4%)  88(31.3%) |
| eCrCl  (mL/min)  < 50  ≥ 50 | 102(40.2%)  112(29.6%) | 23(9.1%)  90(23.8%) | 90(35.4%)  176(46.6%) | 80(31.5%)  167(44.2%) | 62(24.4%)  90(23.8%) | 151(59.5%)  121(32.0%) |
